# Targeting BRAF in thyroid cancer

**DOI:** 10.1038/sj.bjc.6603520

**Published:** 2006-12-19

**Authors:** A V Espinosa, L Porchia, M D Ringel

**Affiliations:** 1 Divisions of Endocrinology and Oncology, Department of Medicine, The Ohio State University College of Medicine and Arthur G. James Comprehensive Cancer Center, Columbus, OH, USA

**Keywords:** papillary thyroid cancer, RET/PTC oncogene, RAS

## Abstract

Activating mutations in the gene encoding BRAF are the most commonly identified oncogenic abnormalities in papillary thyroid cancer. *In vitro* and *in vivo* models have demonstrated that overexpression of activated *BRAF* induces malignant transformation and aggressive tumour behaviour. BRAF and other RAF kinases are frequently activated by other thyroid oncogenes and are important mediators of their biological effects including dedifferentiation and proliferation. Because current therapeutic options for patients with thyroid cancers that are aggressive and/or do not respond to standard therapies are limited, BRAF and its downstream effectors represent attractive therapeutic targets. In this review, data supporting a role for BRAF activation in thyroid cancer development and establishing the potential therapeutic efficacy of BRAF-targeted agents in patients with thyroid cancer will be reviewed.

Thyroid cancer incidence is increasing throughout the world. Although this appears to be due largely to enhanced detection of early thyroid cancers by the more generalised use of thyroid ultrasound and fine-needle aspiration (Davies and Welch, 2006), the number of patients who succumb to this disease is also rising annually (Ries *et al*, 2006). Thyroid cancer therapy is based on a three-pronged approach that includes thyroidectomy, and in more aggressive cases, radioactive iodine therapy and suppression of thyrotropin (TSH) levels using levothyroxine. This regimen achieves outstanding long-term survival rates for most patients with thyroid cancer, particularly for the majority diagnosed with early-stage disease. However, for the subset of patients who do not respond to this treatment paradigm, or who present with aggressive primary tumours, therapeutic options are relatively limited and long-term survival rates are low. Treatment using cytotoxic chemotherapy has been largely unsuccessful and although external beam radiation has been shown to be helpful in palliation, cure is rarely achieved. Therefore, developing new approaches to treating patients with aggressive and/or progressive thyroid cancers is an important avenue of research that requires identification of appropriate molecular therapeutic targets.

Over the past few years, it has become evident that aberrant signalling through the RAS–RAF–MEK cascade is crucial for the development of thyroid cancer. In the case of papillary thyroid cancer (PTC), the subtype that accounts for more than 80% of all thyroid cancers, mutations and/or gene rearrangements in cell surface receptors or signalling molecules in this cascade occur in ∼70% of cases and are tumorigenic *in vitro* and *in vivo* (reviewed in Kondo *et al*, 2006). Of these abnormalities, activating mutations in BRAF (V600E) are particularly common in adults with PTC, accounting for approximately 45% of cases (reviewed in Xing 2005). Because BRAF is activated through multiple mechanisms in the majority of thyroid cancers (see Figure 1), there has been an interest in targeting BRAF for potential therapeutic benefit in patients with thyroid cancer.

## *BRAF* MUTATIONS IN PTC TUMORIGENESIS

**Figure 1:**
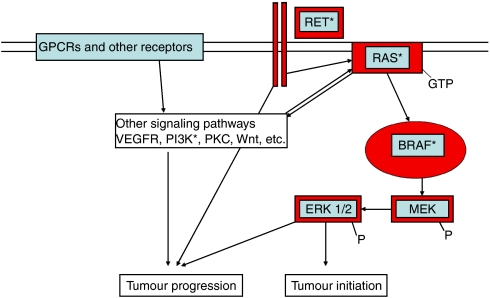
Activation of the RAS–RAF–MEK pathway in thyroid cancer. Constitutive activation via gene mutations or rearrangements in RET, RAS and BRAF (highlighted in red) are the principal initiators of thyroid cancer development through enhanced nuclear translocation of ERK and subsequent transcriptional regulation of target genes. Activation of this tumour initiation pathway occurs in ∼70% of thyroid cancers. Activation of other pathways that cooperatively activate ERK, as well regulate other cell signaling pathways may also be involved with ERK in thyroid cancer progression. ^*^Proteins known to be constitutively activated in thyroid cancer.

The RAF kinases, termed ARAF, BRAF and CRAF, are essential signalling serine–threonine kinase proteins in the RAS-RAF-MEK-ERK cascade (Figure 1). *In vitro* studies in benign thyroid cell models have demonstrated a particularly important role for BRAF, in comparison to other RAF isoforms, as a central regulator of thyroid-specific protein expression (i.e., differentiation) and proliferative capacity (Mitsutake *et al*, 2005a). The discovery that mutations in BRAF resulting in constitutive kinase activation are common occurrences in solid tumours (Davies *et al*, 2002) led a number of groups to analyse thyroid cancers for similar mutations in BRAF. In total, mutations that result in a V600E substitution in BRAF and consequent constitutive activation occur in approximately 45% of PTCs in adults, making BRAF mutations the most common defined genetic abnormality in thyroid cancers (Xing, 2005). In addition to the V600E mutation, rare thyroid tumours have been described with mutations at the 599 and 601 locations that also result in constitutive activation of BRAF kinase (Trovisco *et al*, 2004; Moretti *et al*, 2006E). It has recently been recognised that rearrangements involving BRAF and AKAP9 that result in increased BRAF signalling occur in a small subset of PTCs (Ciampi *et al*, 2005). Thus, taken together, direct oncogenic activation of BRAF is an extremely common event in PTC tumorigenesis.

In addition to BRAF mutations, gene rearrangements resulting in constitutive activation of the tyrosine kinase receptor, RET (RET/PTC oncogenes) that activate the RAS–RAF–MEK and other signalling cascades are common in PTC (Figure 1). These rearrangements, in addition to the AKAP9/BRAF rearrangements and rearrangements involving the NTRK oncogene, are associated with PTCs that develop following radiation exposure. Activation of the RAS–RAF–MEK cascade is not limited to thyroid cancers histologically characterised as PTCs, as activating mutations in RAS genes (particularly N-RAS) occur in 20–30% of follicular thyroid cancers (FTCs) as well as in a smaller percentage of potentially premalignant follicular adenomas (Vasko *et al*, 2003). Interestingly, PTCs that have a follicular growth pattern (follicular variant of PTC) also frequently have activating mutations in RAS oncogenes (Zhu *et al*, 2003; Adeniran *et al*, 2006). Therefore, increased RAF kinase activity induced by activation of upstream signalling molecules events is also common in thyroid cancer, further accentuating the broad importance of this family of signalling molecules in thyroid tumorigenesis.

Functionally, BRAF V600E is capable of inducing thyroid cell transformation *in vitro* and to induce thyroid cancer *in vivo* in transgenic mice with thyroid-specific expression of the protein (Knauf *et al*, 2005). These data confirm that BRAF V600E is an oncogene for thyroid cancer. Because BRAF and the other RAF kinases were putative downstream signal transducing molecules for RET/PTC, and because mutations in BRAF and RAS, and rearrangements in NTRK and RET/PTC are largely mutually exclusive in human PTC samples, it was suggested that the RAS/RAF/MEK pathways might represent a linear cascade whose activation promotes thyroid cancer formation (Kimura *et al*, 2003; Soares *et al*, 2003). To test this hypothesis, a series of elegant gene expression studies were performed using *in vitro* models and clinical samples to determine the role of BRAF in RET/PTC effects in thyroid cells (Melillo *et al*, 2005; Mitsutake *et al*, 2005b) and the degree of overlap in gene expression in tumours with either genetic abnormality (Giordano *et al*, 2005). Taken together, these studies demonstrate that BRAF and RET/PTC share similar mechanisms for their acute effects on thyroid cells and that there is substantial, although not complete overlap in gene expression profiles of PTC expressing either oncogene. The relative importance of BRAF signalling in the actions of the different RET/PTC or RAS oncogenes has not yet been carefully dissected, although it seems likely that BRAF, and perhaps other RAF kinases, play an important role based on data from cell model systems.

Important distinctions between tumours that express RET/PTC vs BRAF V600E are their relationships to radiation exposure and age at PTC diagnosis. Convincing functional and epidemiologic evidence supports the ability of radiation to induce RET/PTC gene rearrangements and a high prevalence of RET/PTC expression in radiation-induced PTCs (reviewed in Ciampi and Nikiforov, 2006). In contrast, BRAF mutations appear to be uncommon in radiation-related PTCs. In addition, RET/PTC is more prevalent in childhood thyroid cancers, whereas BRAF V600E mutations are extremely rare in children and the age of patients with PTCs who express BRAF V600E is more than that of the general PTC population (Xing *et al*, 2005; Ciampi and Nikiforov, 2006).

## BRAF MUTATIONS IN AGGRESSIVE PTC

The relationship between the BRAF V600E mutation and aggressive tumour behaviour is controversial. In several studies, the presence of a BRAF V600E mutation has been associated with a more aggressive clinical course (Namba *et al*, 2003; Nikiforova *et al*, 2003; Xing *et al*, 2005; Riesco-Eizaguirre *et al*, 2006). However, these results have not been confirmed in a number of other studies (Fugazzola *et al*, 2004; Kim *et al*, 2005; Fugazzola *et al*, 2006). This discrepancy may be related to study size, the duration of follow-up, differences in histological designation of tumours, and/or the specific populations analysed. Similarly, although all studies associate the presence of a BRAF V600E mutation with papillary architecture, several studies have reported a higher prevalence of BRAF mutations in tumours classified as tall cell or columnar cell variants of PTC that are associated with more aggressive clinical behaviour and loss of responsiveness to radioiodine (Nikiforova *et al*, 2003; Begum *et al*, 2004). However, as noted above, an association between BRAF mutations and aggressive tumour behaviour has not been demonstrated in all populations, making the clinical relevance of this association uncertain. It is also of interest that the incidence of BRAF V600E mutations in undifferentiated anaplastic thyroid cancer is similar to that in well-differentiated early-stage tumours in several studies (Namba *et al*, 2003; Nikiforova *et al*, 2003; Begum *et al*, 2004; Soares *et al*, 2004; Trovisco *et al*, 2004; Trovisco *et al*, 2005) supporting the concept that some anaplastic thyroid cancers arise from more typical forms of PTC and suggesting that BRAF signalling may be functionally important in anaplastic thyroid cancers. These data also suggest that BRAF mutations may not be independently sufficient to induce dedifferentiation. Finally, it has been proposed that detection of BRAF V600E might be used to stratify patients for more aggressive initial therapy or to help diagnose PTC on indeterminate fine-needle aspiration samples. In fact, methods for rapid detection of this mutation on fine-needle aspiration samples have been developed (Xing *et al*, 2004). However, the true predictive nature of BRAF mutations for aggressive behaviour, particularly when considering the high frequency of BRAF mutations in small PTCs associated with an excellent prognosis, remains uncertain.

The potential mechanisms by which BRAF mutations might predispose PTCs to have a more aggressive course and/or poor responsiveness to standard therapies are not certain. *In vitro* studies in thyroid cells suggest that BRAF V600E induces invasion via increased expression of matrix metalloproteinases (MMP12) (Mesa *et al*, 2006) and that it reduces expression and membrane localisation of the Na, I symporter responsible for thyrocyte uptake of iodine (Riesco-Eizaguirre *et al*, 2006). The thyroid-specific BRAF V600E mouse model also develops invasive and poorly differentiated thyroid cancer that develops progressive local invasion in association with markers of aggressive tumour behaviour (Knauf *et al*, 2005). In human thyroid cancers, it has recently been reported that BRAF V600E is highly associated with (1) overexpression of VEGF and that overexpression of VEGF was highly associated with increasing tumour stage and invasion (Jo *et al*, 2006) and (2) reduced membrane expression of NIS (Riesco-Eizaguirre *et al*, 2006). Thus, a combination of markers of aggressive behaviour, perhaps including BRAF mutation status, may be useful in improving predictions of tumour behaviour. However, it is unclear that any of these markers is more accurate than the currently used clinical staging systems.

In comparison to BRAF mutations that occur at similar frequencies in well-differentiated PTCs and undifferentiated tumours, several events (Figure 1), including mutations in the P53 gene and overactivation of the Wnt-signalling cascade occur much more frequently in undifferentiated thyroid cancers (Kondo *et al*, 2006). Whether enhanced RAF kinase activity influences these later changes is an uncertain but important question when considering BRAF and RAF kinases as therapeutic targets for patients with undifferentiated forms of thyroid cancer.

## BRAF AS A THERAPEUTIC TARGET FOR PTC

### Preclinical studies

Because BRAF V600E expression is common in PTCs, it represents a potentially important target for progressive PTC therapy. This potential may not be limited to just PTCs with BRAF-activating mutations because BRAF and the entire RAF kinase family are activated by other oncogenes involved in thyroid cancer development, in both PTC and follicular carcinomas (Figure 1). A number of compounds have been designed to target RAF kinases at low concentrations, with greater or lesser specificity. Several of these have been tested in thyroid cancer models *in vitro*.

Molecular inhibition of BRAF specifically using siRNA has been shown to inhibit proliferation of several different poorly differentiated thyroid cancer cell lines, some of which express V600E BRAF (Salvatore *et al*, 2006). These data, in concert with the evidence described above that BRAF V600E overexpression increases proliferation and DNA instability of thyroid cells and the central role of BRAF as a critical signalling node in RET/PTC-induced thyroid cell proliferation, suggest BRAF as an appropriate therapeutic target for thyroid cancer.

Sorafenib is a bis-aryl urea (Nexavar, Bayer HealthCare Pharmaceuticals, New Haven, CT, USA) competitive inhibitor of all RAF isoforms, BRAF V600E, and several other tyrosine kinase receptors, including KDR, PDGFR, KIT and RET at nanomolar concentrations *in vitro* (Wilhelm *et al*, 2004). Several studies have demonstrated the ability of this agent to inhibit proliferation of poorly differentiated thyroid cancer cell lines with and without BRAF V600E mutations *in vitro* (Ouyang *et al*, 2006; Salvatore *et al*, 2006). In addition, Sorafenib has been shown to inhibit growth and enhance apoptosis in one anaplastic thyroid cancer cell line in xenograft models at micromolar concentrations that are achievable in patients (Ouyang *et al*, 2006; Salvatore *et al*, 2006). Whether the activity of Sorafenib in thyroid cancer is mediated by inhibiting RAF kinases, RET or its other targets is uncertain. It is of interest that other agents that inhibit VEGF activity specifically have been shown to have salutary effects against thyroid cancer cell lines *in vitro* and *in vivo* (Bauer *et al*, 2003). As described below (see clinical studies section), Sorafenib is currently being evaluated in patients with iodine non-responsive thyroid cancer.

Two additional potent isoquinoline RAF kinase inhibitors, NVP-AAL881-NX and NVP-LBT613-AG-8 (Novartis, Cambridge, MA, USA), have been tested *in vitro* and *in vivo* against a panel of poorly differentiated thyroid cancer cell lines (Ouyang *et al*, 2006). Both agents inhibit proliferation and induce cell death in thyroid cancer cell in micromolar concentrations, although there was variable resistance to these effects among the cell lines. *In vivo*, both agents inhibited xenograft growth, although some toxicity was reported at higher doses with NVP-LBT613-AG-8. Similar to Sorafenib, these compounds inhibit KDR function and it is of interest that the effects *in vivo* did not correlate with inhibition of RAF activity for one of the cell lines, further suggesting that several targets may be therapeutically important in progressive thyroid cancer.

A number of additional compounds targeted to inhibit RAF kinases are in development that vary in their designs and specificity (Thompson and Lyons, 2005). Preclinical data suggest that inhibition of RAF kinase activity, alone or in combination with other effects, may have therapeutic benefit. It should be noted that there are a number of other studies using kinase inhibitors that do not have appreciable effects on RAF kinases. Preclinical and clinical data using these agents are not addressed in this mini review; however, they support the notion that inhibition of thyroid cancer cells at multiple targets may be therapeutically advantageous (Braga-Basaria and Ringel, 2003).

The potential enthusiasm that targeting the linear RAS–RAF–MEK–ERK cascade in patients with thyroid with different oncogenic alterations must be reconciled with the recent data of Solit *et al* (2006) who examined the response of a series of melanoma cell lines to MEK1/2 inhibitors *in vitro*. These authors reported that cell lines with BRAF-activating mutations were much more sensitive to MEK inhibitors in comparison to those with RAS mutations. This finding was generalised to cancer cell lines derived from different tissues, although thyroid cancer cell lines were not included. These data suggest that expression of constitutively activated BRAF uniquely predicts sensitivity to MEK inhibition compared to tumours with RAS mutations, perhaps owing to RAS-mediated activation of signalling molecules not in the linear RAS–RAF cascade (Figure 1). Whether BRAF mutations uniquely predict response to MEK inhibitors in thyroid cancer will need further *in vitro* and *in vivo* study.

### Clinical studies

Until recently, there were very few clinical trials available for patients with iodine nonresponsive forms of thyroid cancer. However, with the expanding development of kinase inhibitors and the success of using a targeted approach to treating other forms of cancer with well-defined genetic causes, such as chronic myelogenous leukaemia and gastrointestinal stromal tumours, thyroid cancer has become an obvious target for drug development. As described above, preclinical studies suggested that tyrosine kinase inhibitors that also block RAF activation might be useful for patients with thyroid cancer. Moreover, these studies suggested broad degrees of activity in thyroid cancer cell lines independent of BRAF V600E mutations due either to activity of the compounds at alternative targets or activation of RAF kinases by other oncogenes (or both).

Of the BRAF-targeted agents, Sorafenib has progressed to approval by the United States Food and Drug Administration for therapy of renal cell carcinoma and is under evaluation for melanoma, thyroid cancer and other malignancies. In phase 1 studies, patients with metastatic solid cancers were treated with different dosing schedules of Sorafenib and one partial response in patients with PTC was reported (Strumberg *et al*, 2002). These data, along with the logic of targeting RAF, RET and VEGF in thyroid cancer therapy led to the development of several phase 2 studies of Sorafenib as a solitary agent for treatment-refractory differentiated or anaplastic thyroid cancer that are ongoing or were recently completed. Side effects of this agent include hypertension, hand–foot syndrome, rash and fatigue that can be dose-related, and in the case of the rash, may be associated with tumour response to the compound (Strumberg *et al*, 2006).

It is of interest that Sorafenib is active against renal cell carcinoma, a tumour that does not frequently have BRAF mutations, suggesting that some of its clinical activity may be directed against non-RAF targets. Sorafenib is being studied in combination with cytotoxic chemotherapeutic agents, such as doxorubicin in melanoma where, despite a high frequency of BRAF mutations, this agent does not appear to be majorly biologically active (Richly *et al*, 2006). Combination therapy has not yet been tested for activity in thyroid cancer *in vitro* or *in vivo*. It seems likely that at least some of the effects of this agent *in vivo* are likely to be due to inhibition of RET, KDR and/or PDGFR.

A preliminary analysis of 16 chemo-naïve patients (of 58 total study participants) with progressive PTC treated with single-agent Sorafenib (400 mg twice daily) in a phase 2 study has revealed a RECIST-determined partial response (>30% reduction) in one of 16 patients, with minor responses (20–30% reduction) in 50% over a mean follow up of 9.9 months (RT Kloos, personal communication). The durability of those responses is variable, with some patients progressing rapidly after initial responses over several months and others developing disease stability for more than 12 months. The final response analysis and the correlations between response and tumour genotype and signalling inhibition on biopsy samples are pending. However, based on these data, it appears that Sorafenib alone will be unlikely to induce complete remissions from thyroid cancer. These results compare favourably to a recently reported phase 2 study of Sorafenib in patients with melanoma in which the response rate was lower and the rate of progression was higher (Eisen *et al*, 2006).

It is important to recognise that the precise mode of action for Sorafenib in thyroid cancers is uncertain. This compound has a number of potential therapeutic targets that may be responsible for its clinical activity. Finally, as noted above, targeting MEK may represent a more potent alternative for tumours with BRAF-activating mutations (Solit *et al*, 2006).

## SUMMARY

Activating mutations in the BRAF gene and constitutive signalling through RAF kinases are common events in the development of thyroid cancer. BRAF V600E mutations may be associated with thyroid cancer progression, have been defined as thyroid oncogenes experimentally, and may be, in part, responsible for tumour dedifferentiation and loss of response to standard therapies, such as TSH suppression and radioiodine. Because patients who do not respond to these therapies have a poor prognosis with no proven effective therapeutic options, they represent an important population for the development of new therapies. BRAF and its downstream signalling pathway represent a potential therapeutic target that is being actively studied in thyroid cancer. The studied compounds vary in their specificity for RAF kinases, but have shown potential benefit in preclinical studies and in early reports of clinical trials. Validating the actual targets of these drugs in clinical trials and exploration of combination therapies are important future goals to maximise the potential utility of this approach for patients with progressive thyroid cancer.
