# Targeting BRAF in thyroid cancer

**DOI:** 10.1038/sj.bjc.6603676

**Published:** 2007-04-17

**Authors:** A V Espinosa, L Porchia, M D Ringel

**Correction to:** *British Journal of Cancer* (2007) **96**, 16–20. doi: 10.1038/sj.bjc.6603520

The authors would like to note that the preliminary results of a phase II clinical trial of sorafenib in patients with metastatic thyroid cancer reported as a personal communication in the manuscript have been previously presented as part of the following two abstract presentations:

Kloos R, Ringel M, Knopp M, Heverhagen J, Hall N Weldy L, Arbogast D, Collamore M, Shah M (2005). Preliminary results of Phase II clinical trial of RAF/VEGF-R kinase inhibitor, BAY 43-9006 (sorafenib), in metastatic thyroid carcinoma. *Thyroid*. 2005 Abstracts from International Thyroid Congress, Vol 15, No. S1:S-22.

Kloos R, Ringel M, Knopp M, Heverhagen J, Rittenberry J, Weldy L, Arbogast D, Collamore M, King M, Young D, Shah M (2006). Significant clinical and biologic activity of RAF/VEGF-R kinase inhibitor BAY-439006 in patients with metastatic papillary thyroid carcinoma (PTC): Updated results of a phase II study. *J Clin Oncol*, 2006 ASCO Annual Meeting Proceedings, Part I, Vol 24, No. 18S:5534.

